# Effect of Adding Pyrolysis Carbon Black (CBp) on Soft Friction and Metal Wear during Mixing

**DOI:** 10.3390/polym14071319

**Published:** 2022-03-24

**Authors:** Yiren Pan, Yi Pan, Zhilin Wang, Shuang Han, Wenwen Han, Huiguang Bian

**Affiliations:** College of Electromechanical Engineering, Qingdao University of Science and Technology, Qingdao 266061, China; pyr@qust.edu.cn (Y.P.); 17806168130@163.com (Y.P.); zhilin_wang2021@163.com (Z.W.); china666@163.com (S.H.); hbhanwenwen@163.com (W.H.)

**Keywords:** CBp, friction and wear, surface activation, mixing process

## Abstract

In the cracking process of waste tires, pyrolysis carbon black (CBp), as a solid product, accounts for about 35% of the total tire rubber content. Here, the treated CBp has been gradually applied to the tire formula to improve the recycling efficiency of waste tires. This study elucidated the influence of adding CBp during the tire mixing process on soft friction and metal wear. Compared with industrial carbon black (I-CB), the friction coefficient of CBp was smaller at different mixing stages, and the ripple caused by adhesion friction was not evident. After the modified CBp (M-CBp) was obtained by implementing the surface activation of common CBp (C-CBp), the friction coefficient between M-CBp and metal increased by 10%, while the filler dispersion and comprehensive mechanical properties showed an upward trend. The wear rate of metal was higher than that observed after adding I-CB during the same mixing mode; thus, it was necessary to strengthen the wear resistance of the inner-wall surface of the mixing chamber. The –OH group on the M-CBp surface can also participate in the silane coupling reaction and aggravate the metal wear of the mixer chamber wall. Through a comparison of results, the mixing friction coefficient can reflect the strength of filler–rubber interaction, which in turn can preliminarily represent the dispersion effect and comprehensive properties, reveal the reason behind the poor performance of CBp, and highlight the need for modification from the perspective of tribology.

## 1. Introduction

Tires, one of the most important products in modern society, promote continuous development of the global transportation industry and economy [1]. Billions of tires are produced globally every year, which has led to a large number of waste tires; thus, treatment and recycling of waste tires have become urgent problems that need to be solved. The comprehensive utilization of waste tires as a valuable renewable resource has an important strategic significance to alleviate the shortage of rubber resources and petrochemical resources [2,3]. At present, waste tire rubbers are disposed of by landfilling, retreading, gasification, incineration, and pyrolysis. As a typical circular economy mode, pyrolysis technology is recognized as the most effective treatment method for waste tires, as it can fully utilize waste tires without harming the environment [4,5]. Moreover, waste tires can be pyrolyzed into oil, gas, cracked carbon black, etc.

As an important industrial raw material, carbon black is widely used in plastics, energy materials, lithium ion battery materials, catalyst materials, and biological sensors [6,7,8,9,10,11]. It plays a particularly important role in the rubber reinforcement process. Due to the current lack of resource consumption, reapplication of the carbon black obtained from the thermal cracking of waste tires to the production of rubber products is an important means by which to save energy and improve recycling. Waste tire pyrolysis carbon black (CBp) is prepared by decomposing the organic matter in the tire in a high-temperature cracking furnace and removing the volatile matter; here, the temperature is controlled between 300–450 °C [12]. The flue gas generated in the cracking process is condensed and separated to obtain gas, oil, and a small amount of water vapor. The steel wire is separated using a magnetic separator. The remaining part is modified, granulated, and dried to obtain cracked carbon black. The entire cracking process is irreversible; the main sources of ash are the accessory ingredients added during the manufacturing process of tires and the catalyst added during the thermal cracking process of different wastes. In previous studies, surface active sites of CBp decreased by one third. The low activity of carbon black leads to poor dispersion in large particle aggregates during the mixing process, thereby resulting in poor compatibility between carbon black and rubber and negatively affecting the comprehensive mechanical properties of regenerated carbon black. Cheng et al. [13] studied the influence of CBp on butadiene rubber (BR) and natural rubber (NR); apart from a slight decrease in the tensile stress, other physical and mechanical properties were not different from those obtained via the reinforcement effect of CaCO_3_, which met the national standards and greatly reduced the cost. However, CBp is directly used as a rubber reinforcing material to replace carbon black with smaller particles that provide a larger specific surface area, and its performance is lower than that of ordinary commercial carbon black. Kim et al. [14] performed an acid pickling treatment on CBp to reduce its ash content and modified CBp by using stearic acid. The reinforcement effect of CBp in carbon black was improved, but it could only achieve semi-reinforced carbon black. Therefore, improving the surface activity of CBp is an important way to improve its applicability or replace normal carbon black.

The mixer is important for improving the dispersion of filler rubber and augmenting the comprehensive quality of filler rubber through mechanical action. Yiren Pan [15] has analyzed the effects of the physical and chemical changes of carbon black at different stages on the friction and wear of an internal mixing chamber wall during the mixing process of carbon black. The conclusions obtained were used to study how to improve the mixing effect and prolong the service life of an internal mixer. Meanwhile, corresponding research is conducted on the residual chemical composition of carbon black and the surface abrasion of an internal mixing chamber wall. To further improve the surface activity of CBp and reduce the surface ash content, an acid pickling process with high-temperature calcination and hydrogen peroxide modification is necessary to produce some chemical residues on the surface or voids of modified CBp [16,17,18,19,20]. However, only a few studies have elucidated the influence of activated CBp on the friction characteristics between the activated CBp and an internal mixing chamber wall, including the wear changes in the wall surface during mixing. Therefore, to prevent the chemical residues on the CBp surface from affecting the wear of an internal mixing chamber and rotor, and thus negatively affecting the mixing quality, the overall influence of modified CBp on the internal mixing chamber is worth studying.

This study focuses on the friction between CBp and an internal mixing chamber wall during mixing and determines the change in the wear of the internal chamber wall surface. Furthermore, it explains the reasons behind the poor dispersion effect and poor performance of modified CBp in previous studies from the perspective of tribology. The effects of replacing industrial carbon black (I-CB) with CBp on the blend properties, friction between rubber and internal mixing chamber wall, and the wear of this wall were compared with regard to the tread compound formula (silica- and carbon black-based formula). Through the analysis of experimental data, tribology is used to improve the mixing effect of CBp, and the results are used for process modification and rotor structure optimization to improve the mixing effect and service life of mixing elements.

## 2. Materials and Methods

### 2.1. Materials

Thailand 20# Standard Rubber (TSR20) is the NR used in this study, Thailand. Polymerized styrene–butadiene rubber (SBR; RC2557S) with a 57% vinyl content, cis-polybutadiene (BR9000), and sulfur (S) products were sourced from PetroChina Dushanzi Petrochemical Company, China. Carbon black (N330) was a product of Cabot, America, while CBp was made by Qingdao University of Science and Technology, Qingdao, China. Silica (Silica115MP) products were sourced from Rodia, while the silane coupling agent (Si69mix) was from Nanjing Shuguang Chemical Group, China. Meanwhile, 1,3-Diphenylguanidine (DPG) and N-cyclohexyl benzothiazole-2-sulphenamide (CZ) were obtained from Guiechem, China. The protective and activation systems are qualified products approved by the industry.

Among them, CBp, made by the Qingdao University of Science and Technology, has been simply activated to study the friction between the CBp compound and mixer chamber wall, as well as the wear of the mixer chamber wall at different stages of the mixing process from the perspective of tribology, in order to help analyze the reasons for the differences in the properties of the CBp compound after replacing I-CB.

**Figure 1:**
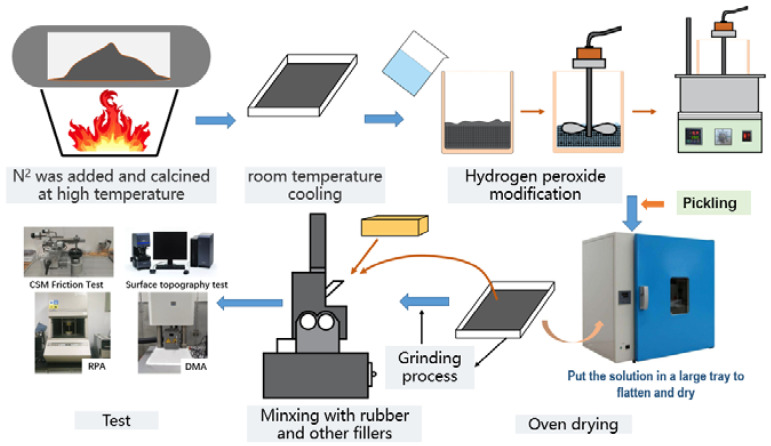
Process of modified CBp.

To study the effect of replacing I-CB with C-CBp and M-CBp in the experiment formula on the friction and wear between the compound and internal mixing chamber wall during the mixing process, M-CBp must be obtained through multiple complex processes by using C-CBp, which was made by the Qingdao University of Science and Technology, to ensure the comparability and accuracy of the experiment. The modification process is shown in Figure 1.

### 2.2. Experiment Methods

1. Friction and wear changes between common CBp and the mixer chamber wall in different mixing stages: To study the friction and wear between C-CBp and the mixer chamber wall materials at different stages in the mixing process, compare the test results with ordinary carbon black. The reason why the performance of cracked carbon black rubber is lower than that of ordinary carbon black rubber is analyzed from the surface morphology of rubber and metal materials before and after friction. The sample formula is TSR20: 50 phr, BR9000: 50 phr, CBp: 58 phr.
2. Influence of replacing I-CB with CBp in different formulations on the friction properties between the compound and mixer chamber metal in the mixing process: C-CBp and M-CBp were replaced with I-CB in the original formula, which was dominated by carbon black or silica, to explore the influence of friction and wear with the mixer chamber wall during mixing. Based on the tribological point of view, the effect of C-CBp and M-CBp replacing I-CB on the friction coefficient between the compound and mixer chamber wall, as well as properties of the compound, were analyzed. By comparing the changes in various properties of rubber to reveal the relationship between the friction coefficient changing and the comprehensive mechanical properties of CB compound. At the same time, the surface wear of the mixing chamber wall was studied after the equivalent amount of C-CBp and M-CBp replaced the I-CB in the original formula in the mixing process. The formulation is shown in Table 1.

### 2.3. Experimental Process

(1) Comprehensive mechanical property test

① A rubber processing analyzer (RPA2000) was used to test the dynamic rheological properties of the compound. Strain scanning of the mixing rubber was performed under the following conditions: frequency of 1 Hz, temperature of 60 °C, and strain range of 0.1 °C; meanwhile, frequency scanning was performed at 60 °C, 7% strain, and 0.01 Hz frequency(Alpha, American). ② Indentation hardness was determined using a durometer (Hardness Shore A)(Wallace, England). ③ The dynamic strain was measured via the dynamic mechanical analysis (DMA). The tensile mode was used for temperature scanning, temperature range was −65–65 °C, frequency was 10 Hz, and heating rate was 2 °C/min (GABOMETER-150, Germany). ④ Tensile properties were determined using the UT-2060 tensile force testing machine produced by Taiwan Ucan Technology (Taiwan Ucan, China).

(2) Friction and wear test

**Figure 2:**
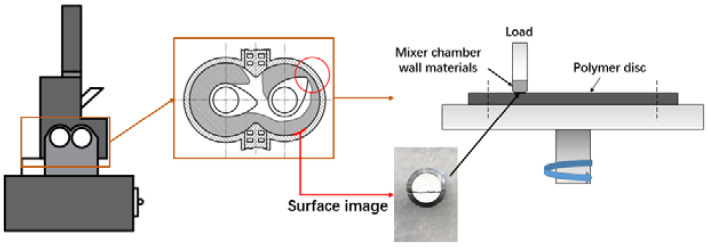
Friction test mode.

According to the contact mode between the rubber and internal mixing chamber wall in the mixing process and based on the principle of force interaction in Newton’s third law, setting the schematic contact mode of the frictional couple is shown in Figure 2. The upper block was metal, the lower disc was rubber compound. To sure the accuracy of the data, a friction test was performed under the same rotation test conditions calculated by the mixing process: a period of 30 min test time, speeds of 60 rad/min, normal loads of 8N, and test temperature is 90 °C. Before each test, the surface metal is wiped with alcohol, and the test points are marked on the metal surface to record the coordinates of the test points, which is convenient for observation of the surface morphology after the friction test. The friction coefficient was continuously recorded by an online data acquisition system attached to the Tribometer of Anton Paar, Austria (short for CSM), and the friction coefficient was the average value during each part of friction test. After the friction test, the worn surfaces data and images of two kinds of disc (compound and mixer chamber wall materials) were examined using Olympus 4500 (Olympus, Japan)to track sliding trajectory, and magnified the test point at the same position with a 20× objective lens, observe the changes of surface morphology before and after friction test, and the surface wear rate of mixer chamber wall material is calculated, the calculation of surface morphology test and wear rate test is shown in Equations (1)–(4).

(1) $$\mathrm{Sa}=\frac{1}{A}\iint_{0}^{A}\left|Z(x,y)dxdy\right|$$

(2) Sp=max z(x,y)

(3) Sv=|max z(x,y)|

Wear rate = V/Fnl (cm^3^/Nm) (4)

where V is the wear volume loss (mm^3^), L is the sliding distance (mm) of very circle, F is the load (N), and n is number of circles.

## 3. Results and Discussion

### 3.1. Friction and Wear Changes between Common CBp and Mixer Chamber Wall in Different Mixing Stages

**Figure 3:**
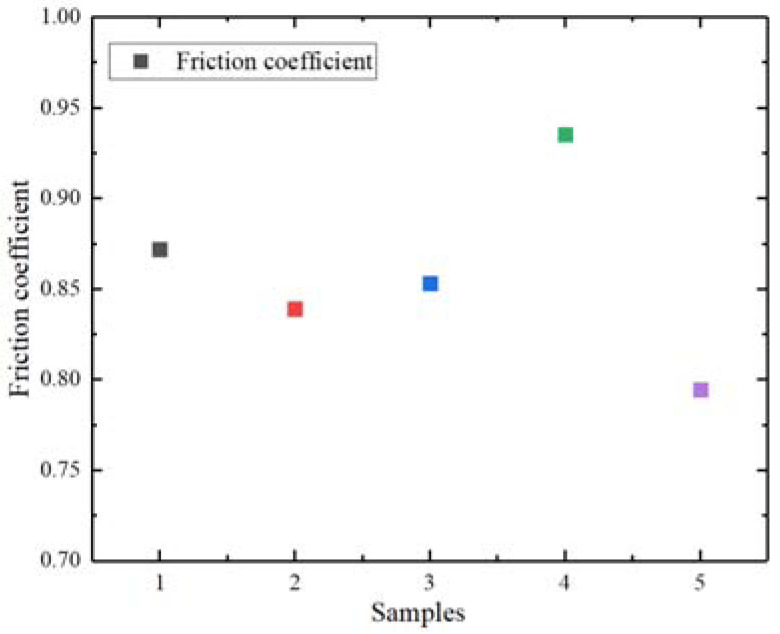
Friction coefficient between CBp and mixer chamber wall at different mixing stages.

Figure 3 shows the change in the friction coefficient between CBp and the mixer chamber wall at different stages of the mixing process, which was determined using CSM. The three main friction modes in the mixing process are adhesion friction, hysteresis friction, and particle friction. The data curve in Figure 2 reveals that the friction coefficient first decreases, then increases, and finally decreases during the mixing process. This trend is mainly observed due to the change in the filling amount, dispersion degree, and viscoelasticity of the compound. After the rubber is plasticized in the internal mixer, the filling amount in the internal mixing chamber increases when the CBp is added; thus, the friction coefficient at the first stage is higher than that at the second and third stages. As the mixing progresses, the carbon black and rubber gradually infiltrate each other; the rubber progressively wraps the carbon black particles, and the filling amount in the internal mixer chamber decreases. In this stage, the CBp aggregate is broken due to the tensile and shear action caused by rotor–rotor interactions and the mixer chamber. Subsequently, the friction between the CBp aggregate particles and the mixer chamber wall gradually weakens. The comprehensive filling amount is reduced, and CBp is wrapped in rubber after crushing; thus, the friction coefficient between the rubber and the internal mixing chamber wall is reduced in Stages 2 and 3. With an increase in mixing temperature, the elasticity of the compound decreases, and the viscosity increases. Although the friction between the CBp aggregate particles and the inner mixing chamber wall decreases, the adhesive friction and lag friction between the CBp aggregate particles and the inner mixing chamber wall increase due to a gradual rise in the rubber viscosity; the combined effect of the three friction modes is the main reason for the sudden increase in the friction coefficient in Stage 4. In the later stages of mixing, CBp is uniformly dispersed and distributed in the rubber. At this point, the CBp particles have been fully wrapped in the rubber and form rubber, leading to the formation of CBp blends. The internal filling amount of the mixing chamber is further reduced; thus, the friction coefficient decreases to its minimum value and becomes stable. The friction coefficient between CBp and the mixer chamber wall in the mixing process is significantly lower than that observed in our previous research.

**Figure 4:**
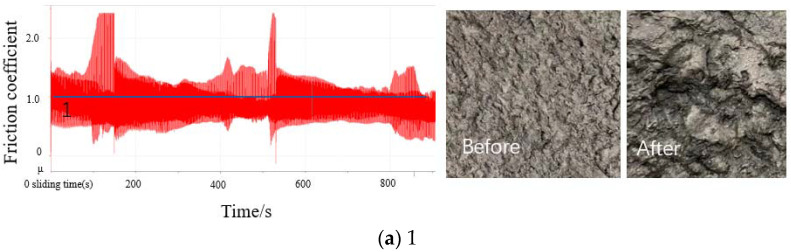

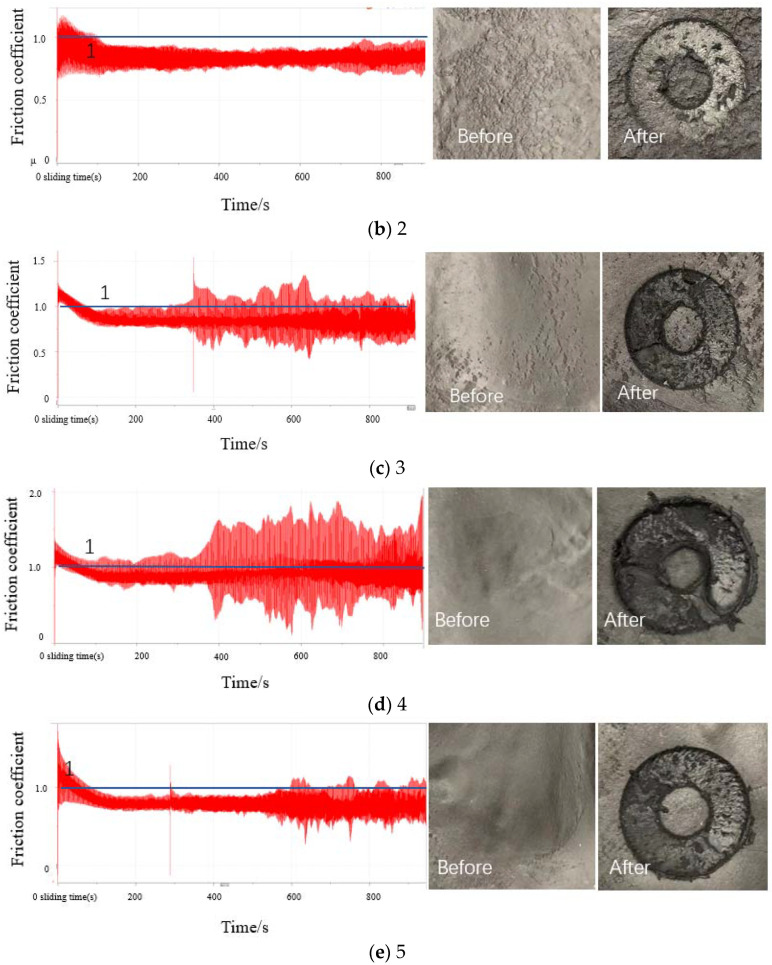
Comparison of friction coefficient curves and changes in the surface of CBp before and after the friction test at different mixing stages.

Figure 4 shows the change in the friction coefficient curve between the CBp and mixer chamber wall, while demonstrating the changes in the CBp surface at different mixing stages. During the friction test, the friction coefficient between the CBp and mixer chamber wall is relatively stable; however, it tends to be high at first and then decreases steadily. We believe that this stationary phenomenon is related to residual ash and CBp surface activity. Even if the CBp had been ground and surface-activated in the experiment, there would still be residual ash on the surface and pores of the CBp. The main components of this residual ash are inorganic salts and zinc oxide (ZnO). In particular, residual ZnO on the CBp surface has been used for powder lubrication in the mixing process. Therefore, during the friction process between CBp and the mixer chamber wall, the residual ZnO in the pores is released through the wedge-shaped gap, and the friction coefficient decreases. By observing the rubber surface after the friction test, it is revealed that the rubber peel shape was similar to the flake peel shape, especially for the surfaces of Samples 3 and 4; this result clearly shows that the ash content on the CBp surface reduced the required friction effect during the mixing process in the mixer chamber, resulting in a poor bonding effect between the CBp and rubber, thus affecting the dispersion of CBp. In Samples 3, 4, and 5, although the viscosity of the compound gradually increases at this stage of mixing, there is no evident curling of large-particle rubber due to adhesive friction during the friction experiment; thus, only adhesive corrugated rubber is observed.

Compared with the friction coefficient of I-CB (studied by Yiren), the friction coefficient between C-CBp and the mixer chamber wall is significantly lower; this is mainly because, during the mixing process, the surface activity and molecular chain sliding theory associated with carbon black are important factors to improve the carbon black dispersion, viscoelasticity of the compound, and friction required during the mixing process. Based on the double shell structure and molecular chain sliding theory of carbon black, the rubber molecular chain forms a bonded rubber unit due to the addition of carbon black; the network formed by this unit functions as the skeleton, connects the free movement area and cross-linking area of the rubber molecular chain, and forms the carbon black–rubber system. Due to the uneven surface activity of carbon black particles, there are active points and adsorption points with different energies, and the rubber molecular chain is adsorbed on the carbon black surface by different forces. In the mixing process, the rubber molecular chain slides on the surface of carbon black particles; consequently, the original chain segment is slowly elongated and straightened under the squeezing, tension, and shear actions of the rotor and the mixing chamber. With an increase in stress, the rubber molecular chain is continuously oriented, and the stress is redistributed on the chain segment to protect the rubber molecular chain of carbon black from fracture due to stress concentration. The chain segment rearrangement, which is caused by the breaking of the carbon black aggregate to form a new surface, will occur continuously during the mixing process. After infinite cycles, the thermal motion will cause the molecular chain to be redistributed between carbon black particles and achieve a new balance. At this time, the length ratio of the rubber molecular chain is relatively uniform, and the friction coefficient gradually reaches a stable level. However, the weak surface activity of CBp is accompanied by the presence of ash and rubber hydrocarbons on the surface, resulting in weak bonding between rubber and carbon black and a low friction coefficient. The reasons behind the poor mixing effect and CBp performance in the mixing process can be revealed from the perspective of tribology. Therefore, we believe that the friction coefficient can be used to determine the surface activity of the filler and the interaction with the rubber molecular chain. Analyzing the friction coefficient curve and rubber shape at different stages shows that the friction coefficient can be used to reflect the filler activity, reveal the interaction strength between the filler and rubber, and control the mixing process.

**Figure 5:**
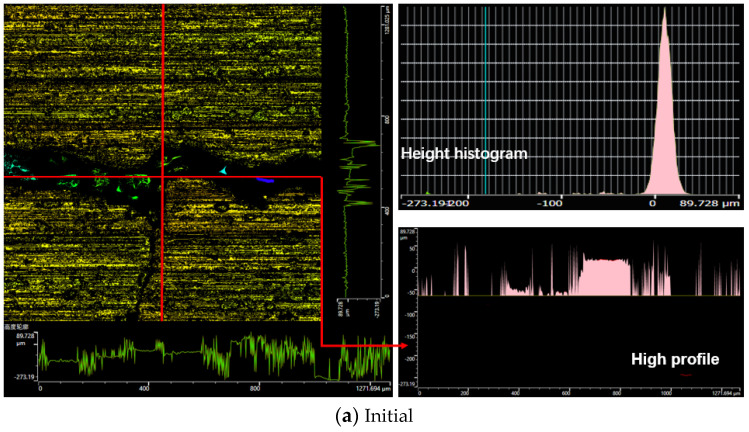

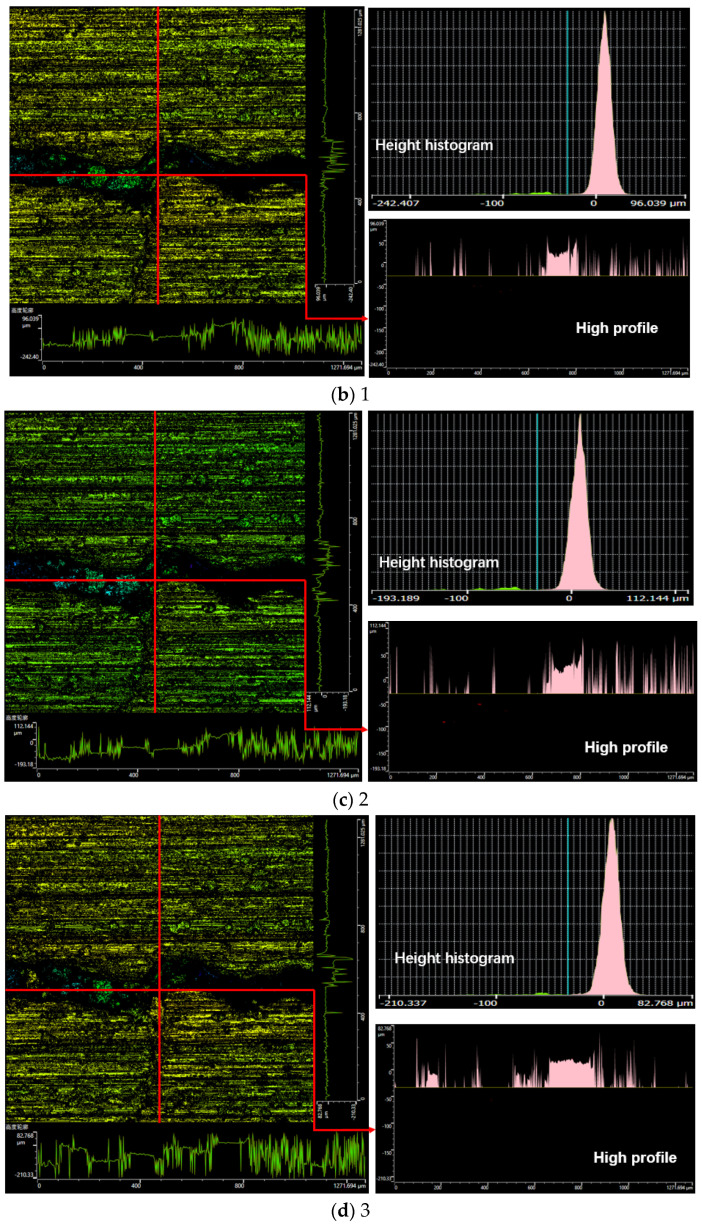

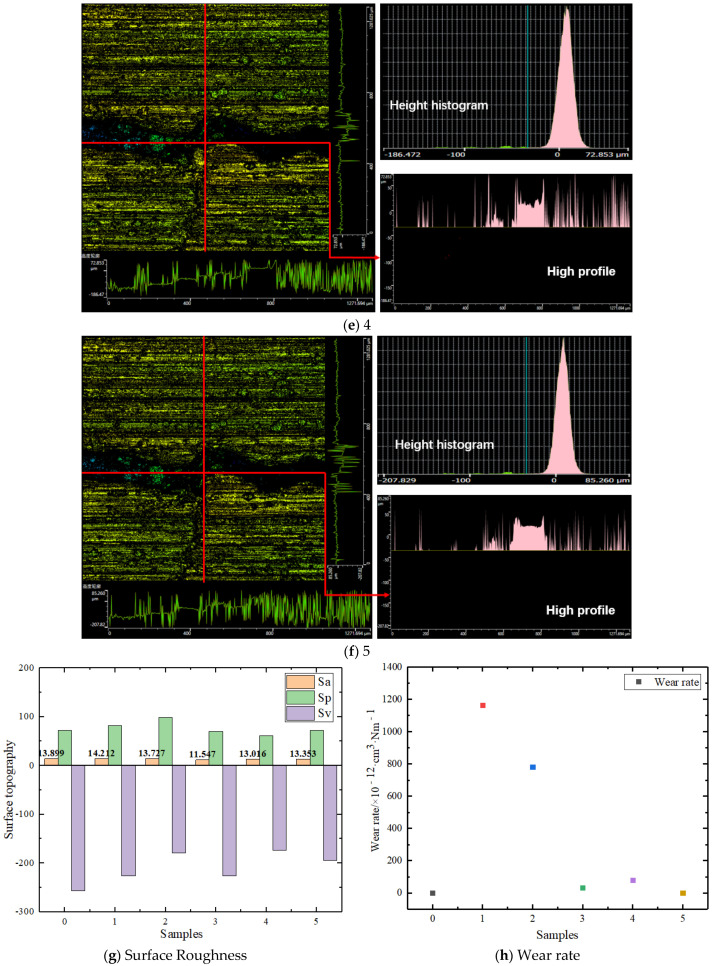
Changes in the surface morphology and data curve representing the internal mixing chamber wall at different mixing stages.

Figure 5 shows the surface morphology changes and data curve associated with the mixer chamber wall at different mixing stages. There is no correlation between the wear of the mixer chamber wall and the friction coefficient between CBp and the mixer chamber wall; the same result is observed for industrial carbon black. Furthermore, due to the friction between CBp and the mixer chamber wall, the surface wear of the wall changes significantly during the first two mixing stages; the wear gradually becomes stable as the mixing continues. The two main reasons behind the increase in wear during the first two stages of the mixing process are: (1) the sudden increase in the internal filling of the mixing chamber and (2) the agglomeration that occurs due to the existence of ash and rubber hydrocarbons on the CBp surface, small particle size, and large specific surface area, resulting in weak binding between the regenerated carbon black and rubber. Consequently, the filler and CBp aggregate accumulate at the inlet of the wedge-shaped gap between the rotor and the mixer chamber wall. As the rotor moves, the aggregate is broken and pressed into the wedge-shaped gap, which in turn aggravates the surface friction of the mixer chamber wall (Figure 3). Due to continuous mixing, the aggregate is gradually broken. Under the chemical action of other fillers, the aggregate gradually infiltrates the rubber, and the surface wear of the mixer chamber wall is reduced.

**Figure 6:**
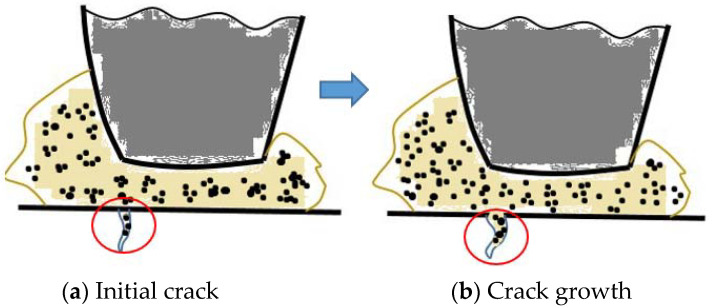
Crack growth process of mixer chamber wall.

We also paid attention to the changes in the metal surface cracks on the mixer chamber wall. By observing the three-dimensional morphology image of the mixer chamber wall in Figure 5a–f, it is found that in the progress of mixing, flaky wear and spalling will occur at the edge of the surface crack, and the morphology and height of the crack also change in real time. Compared with the characteristics of common industrial carbon black and CBp, the main reasons for the surface crack propagation of the internal mixing chamber wall after the friction between the CBp compound and the mixer chamber wall are as follows: Due to the high ash content on the surface of CBp, CBp will be used in rubber products after modification after improving its surface activity. Generally, CBp uses a hydrogen peroxide alkali solution to improve its activity, and then is neutralized with acid solution. However, such a treatment process will lead to residual acid on the surface of the CBp, which will cause corrosion wear on the inner wall of mixer chamber, especially the inner wall of a crack. Because there is some residual ash on the surface of the CBp and the particle size is small, the bonding performance between CBp and rubber is slightly low, even though the modification was performed to improve the CBp activity and achieve a semi-reinforcing effect. When the rubber enters the wedge-shaped space formed by the rotor and the mixer chamber wall, under the pressure of the rotor, a small content of CBp compound and CBp aggregate particles will be pressed into the crack, as shown in Figure 6a. Based on the rubber surface image after the friction test in Figure 4, it was found that the presence of ash on the CBp surface reduces the viscosity of the polymer. Therefore, it is difficult to retrieve the CBp compound and CBp aggregate particles from the crack through the viscoelasticity of the rubber itself in the mixing process. At the same time, the transported CBp compound and CBp aggregate particles rub with the inner wall of the crack, resulting in flake spalling of the metal convex surface on the inner wall of the crack. As the mixing continues, more and more CBp compound and CBp aggregate particles remain in the cracks, resulting in the longitudinal growth of internal mixing chamber wall cracks and the deepening of surface cracks, as shown in Figure 6b.

### 3.2. Influence of Replacing I-CB with CBp in Different Formulations on the Friction Properties between the Compound and Mixer Chamber Metal in the Mixing Process

#### 3.2.1. Comparison of SEM Morphology of Three Kinds of Carbon Black

**Figure 7:**
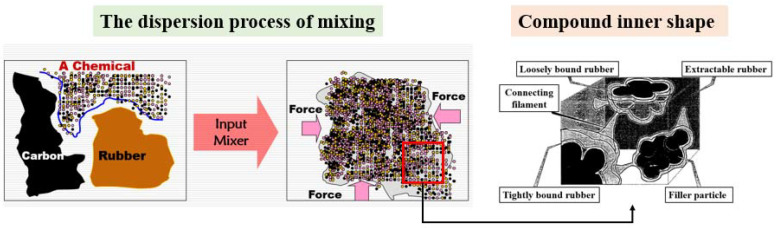
Mixing process and internal shape of the obtained rubber compound.

During the mixing process, carbon black is uniformly dispersed and distributed in rubber due to the shear and tensile action of the rotor and mixing chamber (Figure 7). The intermolecular force of carbon black causes the dispersion in rubber to form small particle aggregates. Meanwhile, after the porosity of the carbon black’s surface increases, it becomes difficult for rubber macromolecules to enter these microspores; this is equivalent to a reduction in the effective area in contact with the macromolecules and a decrease in the interaction between the carbon black and rubber. However, this reduction trend is more evident for the friction coefficient and metal wear between the rubber and internal mixing chamber wall. Therefore, we first studied the particle state of I-CB, common CBp, and activated carbon black.

**Figure 8:**
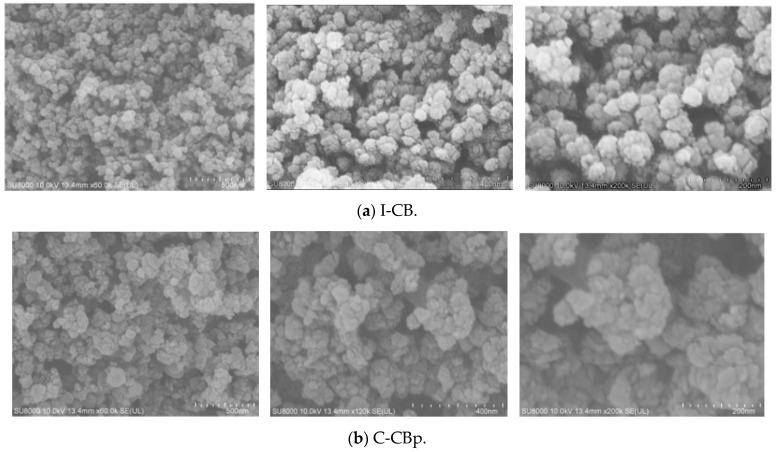

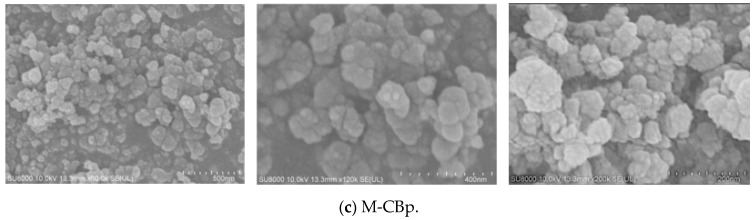
SEM images of three kinds of carbon black.

Comparing SEM images of the three kinds of carbon black reveals that I-CBp and M-CBp have distinct particles and few large aggregate particles (Figure 8). However, there are numerous cracked carbon black aggregates with irregular shapes, indicating that the interaction between carbon black and rubber is strong. The shape of these particles is not conducive to their combination with the rubber molecular chain and decreases the rubber’s viscoelasticity. Due to the weak tensile shear between the rotor and the internal mixing chamber wall, the effective adhesion and lagging friction required in the mixing process reduces; consequently, the friction coefficient between rubber and metal also reduces, resulting in poor mixing.

#### 3.2.2. Changes of Friction and Wear during Mixing and the Relationship between Friction Coefficient and Physical Properties

The influence of CBp on the friction coefficient in the actual mixing process has been elucidated in this study, and the comprehensive properties of different compounds are controlled and monitored through tribological research.

**Figure 9:**
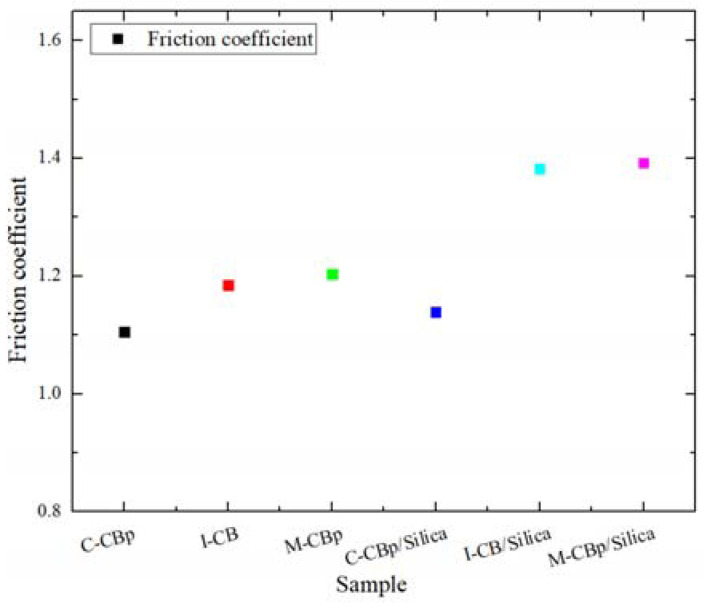
Friction coefficients after I-CB is replaced with two kinds of CBp.

Figure 9 compares the friction coefficient between different compounds and the mixer chamber wall after the carbon black was replaced by CBp. The results showed that, in the carbon black system, when CBp (C-CBp or M-CBp) replaces I-CB, the friction coefficient between the compound and the mixer chamber metal decreases; meanwhile, the friction coefficient of the M-CBp was higher than that of the C-CBp. In the system dominated by silica, the friction coefficients of I-CB/silica and M-CBp/silica with the metal of the internal mixing chamber wall were higher than that of the C-CBp. According to the experimental results of the carbon black system, pyrolysis residual oil, ash, and rubber hydrocarbons were present on the C-CBp surface after a simple treatment, thereby producing a weak carbon black–rubber network. Furthermore, CBp particles are more likely to agglomerate with each other; at this time, particle friction is the most dominant friction. The CBp contacted with the rubber, and thus its friction coefficient reduced. After the C-CBp was modified and activated to obtain M-CBp, the surface residue decreased, and the surface activity increased. Carbon black and rubber form a relatively strong carbon black–rubber network, which increases the viscoelasticity of the rubber, adhesion, and hysteresis friction; thus, the friction coefficient increases. Although the surface activity of M-CBp increases after treatment, a mutual attraction exists between particles; thus, the comprehensive friction coefficient increases. According to the experimental results of the silica system, there will always be a certain amount of hydroxyl (–OH) on the CBp surface after tire pyrolysis due to the relatively complex formula of the recovered tire. When M-CBp is incorporated into the formula of white carbon black, the residual –OH on the M-CBp surface undergoes a silane coupling reaction in the presence of a silane coupling agent; consequently, the rubber viscosity increases, thus augmenting the adhesion and hysteresis friction that impacted the comprehensive friction coefficient. To explore the relationship between the change in the friction coefficient and the comprehensive properties of different compounds after CBp replaced I-CB, the properties of CBp and original carbon black were compared.

**Figure 10:**
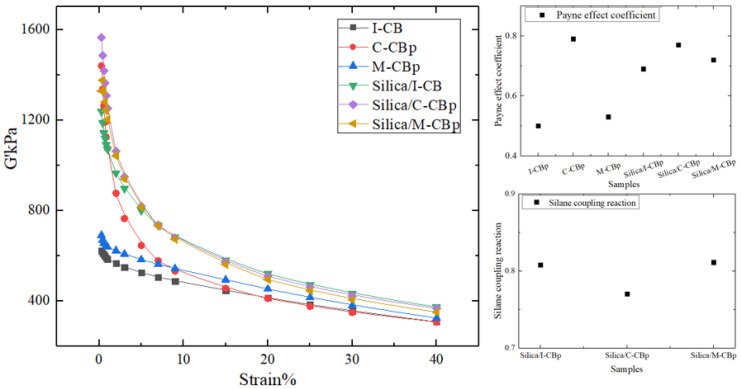
Comparison of Payne effect and silane coupling reaction.

Figure 10 compares the Payne effect and silane coupling reaction of I-CB with those of C-CBp and M-CBp. Overall, there are two main action mechanisms of carbon black or white carbon black in rubber, namely: filler–filler and filler–rubber molecular chain. Therefore, breaking the filler–filler network under the action of deformation is regarded as the Payne effect, and the dispersion effect of filler is a characteristic of the Payne effect. In the carbon black-based system, the Payne effect decreases after replacing I-CB with CBp, which implies that the CBp was poorly dispersed in the mixing process and has a weak binding ability with rubber. After using M-CBp to replace I-CB in the formula, the dispersion effect is significantly higher than that of ordinary CBp due to the increased surface activity. This phenomenon is also observed for the silica-dominant formula. Consequently, it is found that the change in the friction coefficient is consistent with the trend of the Payne effect, which shows that the dispersion degree of carbon black affects the shape of the carbon black rubber network, and thus affects the friction coefficient between rubber and the internal mixing chamber wall during the mixing process.

For the silica formula, the degree coefficient of the silane coupling reaction is calculated through the data obtained from the strain scanning curve (derived from RPA2000). Among them, the degree coefficient of the silylation reaction of the silica comprising CBp is 4.7% lower than that of the silica comprising normal carbon black. When M-CBp replaces C-CBp, the degree coefficient of the silane coupling reaction is increased by 0.41% for the same mixing process; this may be because –OH on the M-CBp surface is involved in the silane Europeanization reaction. Interestingly, the silane coupling reaction has the same trend as that of the friction coefficient of the three compounds. We believe that the change in –OH content affects the degree coefficient of the silane coupling reaction, which in turn influences the change in friction behavior between the rubber and internal mixing chamber wall during mixing. However, the change in friction coefficient can not only elucidate the dispersion degree of the filler, but also indirectly reveal the degree coefficient of the silane coupling reaction under different formulations.

Table 2 shows the vulcanization test data. Among them, MH-ML represents the crosslinking density of the vulcanization network. Due to the existence of ash on the CBp surface, the activity is low. After vulcanization, the crosslinking network density becomes lower than that of normal carbon black. However, this phenomenon shows an opposite trend in silica for the following reasons: (1) When the normal carbon black in the white carbon black formula is replaced with CBp, there will be a certain number of –OH groups on the CBp surface because the cracked tires at this stage are all-steel radial tires and semi-steel radial tires. The silane coupling agent incorporated into the silica not only has a chemical reaction with the –OH group on the silica surface, but also with –OH on the CBp surface. After vulcanization, the rubber molecular chain is connected with the carbon black-vulcanization system; thus, the crosslinking density increases. (2) According to the analysis of the friction coefficient between the silica compound and the internal mixing chamber wall during the mixing process, the compound viscosity is high due to the silica characteristics; this viscosity increases the adhesion and friction between the compound and the internal mixing chamber wall. When rubber is subjected to tensile shear action through the gap between the rotor and the internal mixing chamber wall, this viscosity tends to prevent the dispersion of silica and the silane coupling reaction. When C-CBp replaces I-CB, the residual ash on the C-CBp surface with low activity plays the role of the filling lubricant in the mixing process, which can reduce the rubber viscosity, improve the dispersion effect of silica, and augment the crosslinking density during vulcanization.

Meanwhile, the positive vulcanization time (T90) of the compound increases after the addition of C-CBp and M-CBp due to the –OH groups on the CBp surface. Compared to C-CBp, the addition of M-CBp improves the comprehensive performance of the compound. Furthermore, the change in the friction coefficient after replacing the filler can roughly predict the comprehensive performance of the compound under the same system.

**Figure 11:**
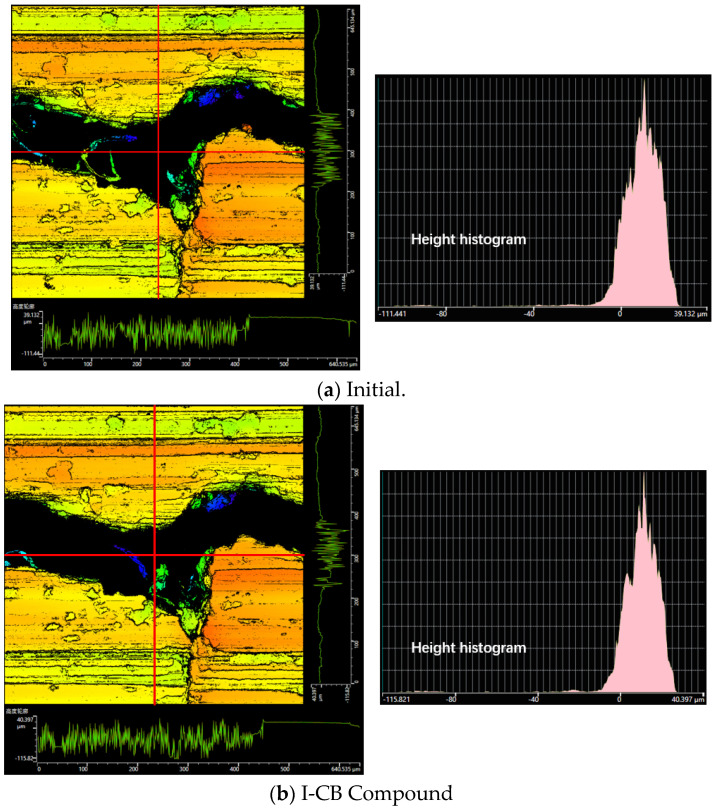

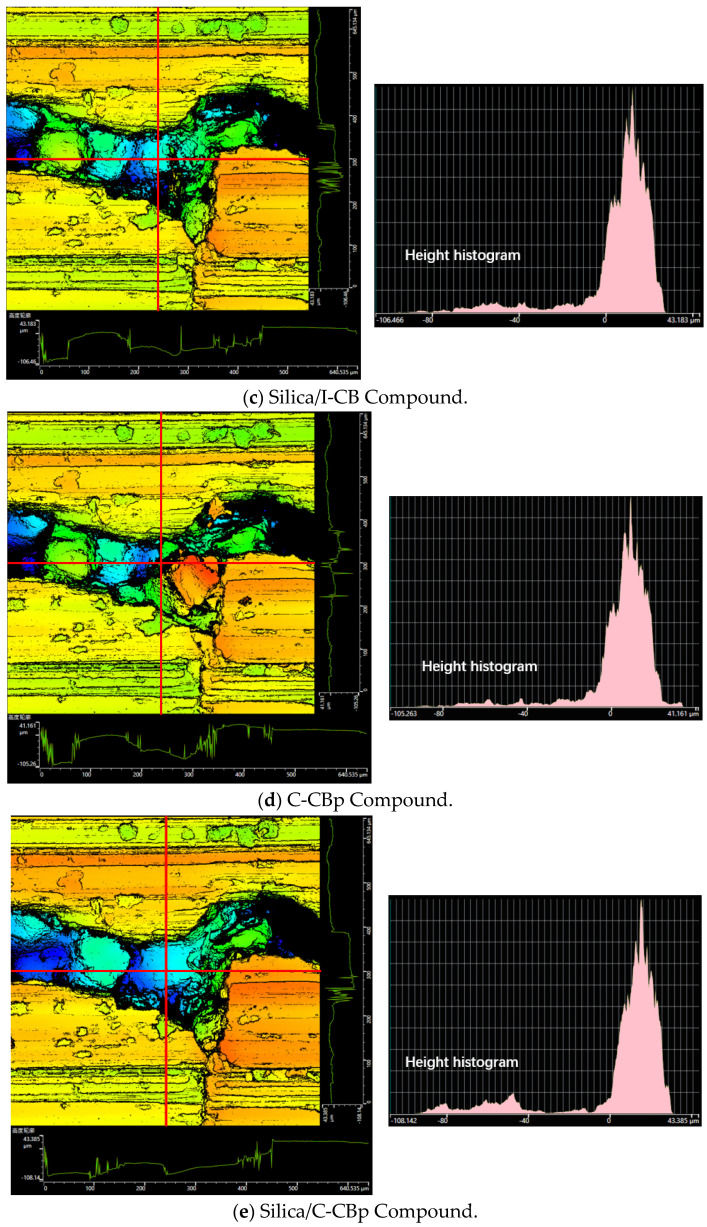

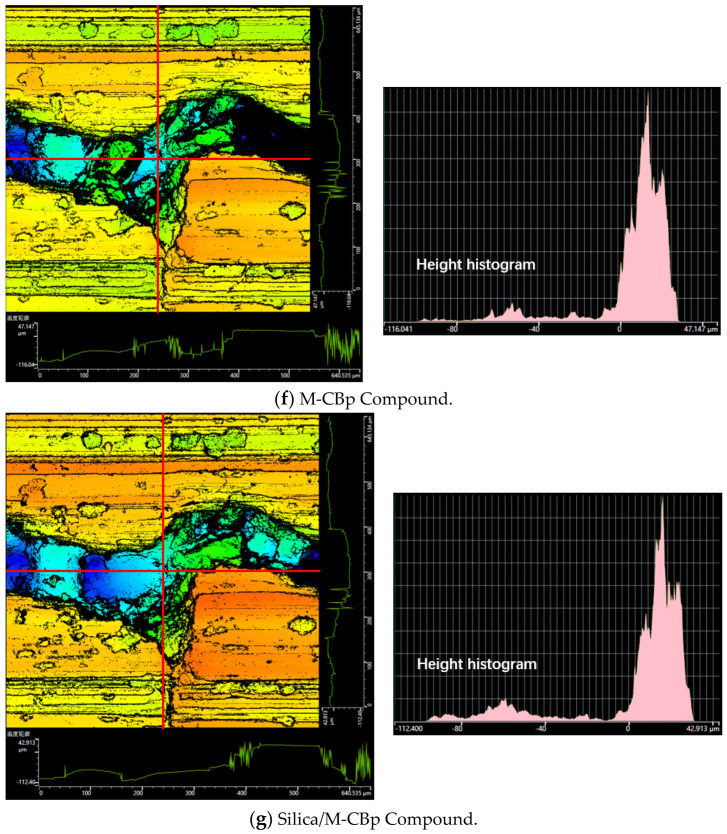
Morphological changes in metal surface before and after friction.

**Figure 12:**
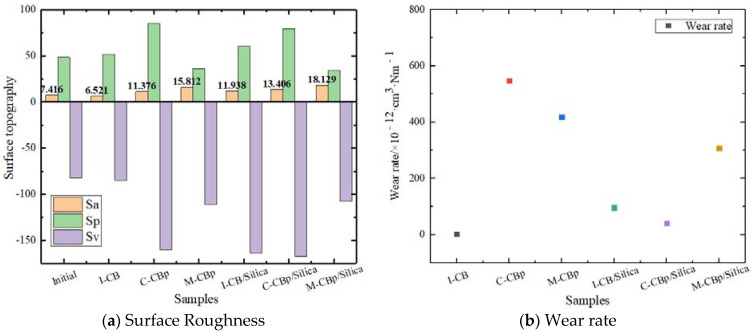
Data curve representing the morphology changes in metal surface before and after friction.

Figure 11 shows the morphological changes in the metal surface before and after friction, and Figure 12 shows the data curve associated with the morphological changes in the metal surface before and after friction. In the carbon black formula, after an equal amount of C-CBp is replaced with I-CB, the change in the wear amount increases according to the data point diagram. After observing the surface crack as a three-dimensional morphology, it was found that the amount of C-CBp in the metal surface crack was significantly more than that of the I-CB pressed into the crack during the mixing process. The surface crack of the mixer chamber wall showed signs of growth, and metal flake peeling occurred around the crack. Since M-CBp continuously contacts with the mixer chamber wall, the residual rubber content in the cracks decreases. Based on the characteristic analysis of the three kinds of carbon black, after I-CB is added to rubber, more carbon black–rubber links are formed, and the viscosity increases due to the small specific surface area and high surface activity of I-CB. Therefore, the adhesive friction and lagging friction between the compound and the mixer chamber metal are high. When the compound enters the wedge-shaped gap between the rotor and the mixer chamber wall, it squeezes into the metal surface crack under pressure. Under the comprehensive action of compound viscoelasticity, the compound and fillers in the crack can be adhered and pulled out after the new compound enters the wedge-shaped space, which can prevent the rubber from further penetrating into the crack and avoid crack growth. The mechanism of M-CBp is similar to that of I-CB. Meanwhile, its surface activity is lower than that of I-CB; thus, it acted as a reinforcing agent and filler in the mixing process. As C-CBp undergoes acid pickling and neutralization during activation, residual acid tends to be on the M-CBp surface. This is also the main reason why the wear of the M-CBp was greater than that of the I-CB.

By observing the changes in metal surface morphology due to friction between the silica compound and the mixer chamber wall metal, it is revealed that, after same amount of C-CBp replaces I-CB, the wear rate and the compound content inside the crack decrease; however, when M-CBp replaces I-CB, the amount of compound pressed into the crack increases after mixing, which is in complete contrast to the experimental results of the carbon black system. Meanwhile, the silica surface tends to be covered with the silane alcohol group, exhibiting high polarity and active chemical properties. It exhibits poor compatibility with general polymers, and, thus, it is easier to form a silica–silica network with high rigidity. Meanwhile, the size of silica particles is small, which can easily agglomerate and enter the surface crack of the mixer chamber. Due to the attraction properties of the silica network, the silica used in the mixing process exhibits high viscosity. Most of the silica aggregates and silica compounds in the gap are taken out by adding a new compound that enters the wedge gap due to its adhesive force. The part of the compound that is taken out tends to rub with the crack wall, resulting in flake peeling. Similarly, during the mixing process, the internal chamber wall is subjected to rigid friction caused by particle aggregates and soft friction (adhesive/lagging) due to compound viscosity, thus resulting in irregular surface wear and plate cracks. Meanwhile, the silica and compound remaining in the crack gap are pressed deeper due to the pressure imposed by the new compound entering the gap. This is the main reason for the increased and faster growth of metal surface cracks during the mixing process of the silica compound. However, when C-CBp is used to replace I-CB in the silica system, C-CBp is used for powder lubrication due to the existence of surface ash impurities (zinc oxide, rubber hydrocarbon, etc.); this can reduce the adhesion and friction between the compound and metal, and form an improved silica–rubber inner network to reduce the amount of compound entering the gap. The addition principle of the M-CBp is the same as that of the I-CB; however, the M-CBp surface comprises more –OH groups than the I-CB surface and less ash impurities than the C-CBp surface. M-CBp also participates in the silane coupling reaction performed using a silane coupling agent, which increases the production of alcohol and water vapor and accelerates the surface wear of the internal mixing chamber wall.

According to the research data, there is no positive correlation between the soft and hard friction coefficients of the mixer chamber wall and the surface wear of the internal chamber wall.

## 4. Conclusions

This study elucidated the effect of replacing I-CB with CBp on the friction and internal chamber wear during the mixing process. Through a variety of experiments conducted on the three carbon black types with different reinforcement systems, the following conclusion were obtained: (1) Compared to the I-CB compound, the friction coefficient between the C-CBp compound and mixer chamber wall at different stages of the mixing process was significantly low; the compound could easily enter the surface crack in the mixing chamber wall. Due to the large amount of surface impurities, CBp was easily agglomerated; thus, the wear rate was high during the early mixing stage. (2) The CBp surface was modified via high-temperature calcination, hydrogen peroxide treatment, and acid neutralization. After improving the surface activity, it was found that replacing I-CB with M-CBp in the carbon black system led to an upward trend for the friction coefficient; the wear rate of the mixer chamber wall wear showed a downward trend, while the amounts of filler and compound entering the crack of the mixer chamber wall were reduced after mixing. In the system dominated by silica, C-CBp can play a lubricating role. The –OH group on the surface of the activated M-CBp participated in the silane coupling reaction, which aggravated the corrosion wear of the mixer chamber wall. (3) After studying the friction and wear between the compound and mixer chamber wall by using three carbon black types during the mixing process, it was found that the dispersion degree and comprehensive performance of rubber are improved when the friction coefficient is high during the mixing process. The high and low values of the friction coefficient during the mixing process represented the interaction strength between the filler and rubber, which was finally reflected in the comprehensive performance of the compound. Therefore, the performance of the compound under the same system could be preliminarily predicted by changing the friction coefficient during the mixing process.

## Figures and Tables

**Table 1:** Sample Formula (Unit: phr).

Raw Material	A	B	C	D	E	F
BR9000	25.5	25.5	25.5	25.5	25.5	25.5
RC2557S	81.81	81.81	81.81	81.81	81.81	81.81
TSR20	15	15	15	15	15	15
I-CB	70	/	/	25	/	/
C-CBp	/	70	/	/	25	/
M-CBp	/	/	70	/	/	25
Silica115MP	/	/	/	45	45	45
Si69mix	/	/	/	6	6	6
DPG	1	1	1	0.8	0.8	0.8
S	1.3	1.3	1.3	1.3	1.3	1.3
CZ	1.8	1.8	1.8	1.8	1.8	1.8
Others		Protection system: 3.5 phr; activation system: 4 phr				

**Table 2:** Vulcanization test data.

List	I-CB	C-CBp	M-CBp	I-CB/Silica	C-CBp/Silica	M-CBp/Silica
ML/dNm	2.42	2.42	2.38	3.33	3.74	3.56
MH/dNm	13.77	11.53	13.57	14.25	15.8	15.15
MH-ML/dNm	11.35	9.11	11.19	10.92	12.06	11.59
TS2/min	5.43	8.88	5.65	5.87	5.48	5.56
T10/min	4.27	7.51	4.56	1.67	1.86	1.74
T50/min	7.04	10.47	8.63	14.29	15.2	14.89
T90/min	12.87	15.7	14.34	28.07	33.38	29.45
100% Tensile Modulus/MPa	3.84	3.65	3.58	3.51	3.68	3.71
300% Tensile Modulus/MPa	8.11	4.64	7.13	5.26	5.35	5.32
Tensile Strength/MPa	15.89	6.79	20.82	17.38	13.82	18.56
Abrasion/%	0.127	0.325	0.130	0.116	0.2377	0.195

## Data Availability

Not applicable.
